# Natural history of hip instability in infants (without subluxation or dislocation): a three year follow-up

**DOI:** 10.1186/1471-2474-15-355

**Published:** 2014-10-28

**Authors:** Blazej Pruszczynski, H Theodore Harcke, Laurens Holmes, J Richard Bowen

**Affiliations:** Department of Orthopedics and Pediatric Orthopedics, Medical University of Lodz, 75 Drewnowska str, 91-002 Lodz, Poland; Department of Medical Imaging, Nemours/Alfred I. duPont Hospital for Children, 1600 Rockland Rd, Wilmington, DE 19803 USA; Department of Epidemiology, Nemours/Alfred I. duPont Hospital for Children, 1600 Rockland Rd, 19803 Wilmington, DE USA; Department of Orthopedics, Nemours/Alfred I. duPont Hospital for Children, 1600 Rockland Rd, Wilmington, DE 19803 USA

**Keywords:** DDH, Hip instability, Hip treatment, Hip dysplasia, Natural history

## Abstract

**Background:**

The natural history of hip instability (without subluxation or dislocation) and treatment in infants remain controversial. We performed a retrospective cohort case-only study with blinded, prospectively collected data to assess normalization of the acetabular index in consecutive untreated infant hips with sonography instability.

**Methods:**

Consecutive hips meeting inclusion criteria were followed by sonography/radiography and data analyzed using tabular and regression models.

**Results:**

In 48 hips, acetabular index measured by radiography normalized within 3 years of age without treatment. Normalization by age occurred: 7 months in 35%, 12 months in 67%, 18 months in 75%, 24 months in 81%, and 36 months in 100%. Two patterns of normalization of the acetabular index were observed: group I showed ossification in a physiological range of normal by 7 months of age, and group II had delayed ossification with later normalization of the acetabular index measurement. Breech presentation (*p* =0.013) and cesarean delivery (*p* =0.004) statistically directly correlated with a later normalization.

**Conclusions:**

The natural history of infant hip instability (without subluxation or dislocation), which is reduced at rest and unstable with stress as diagnosed by the Harcke method of sonography, has spontaneous normalization of the acetabular index within 3 years of age. We suggest three patterns of acetabular ossification in unstable infants’ hips: (I) normal ossification, (II) delayed ossification with normalization of the acetabular index by age 3 years, and (III) defective secondary centers of ossification with an upward tilt of the lateral acetabular rim in adolescence.

**Electronic supplementary material:**

The online version of this article (doi:10.1186/1471-2474-15-355) contains supplementary material, which is available to authorized users.

## Background

Developmental dysplasia of the hip (DDH) is an abnormality of the hip with pathologic alteration in size, shape, and organization of cells [1] and is manifested by both soft tissue (stability) and bony (acetabular) components. Prior to sonography, the infant hip was assessed clinically and confirmed radiographically. Sonography by the Harcke method evaluates the hip joint for soft tissue stability and bone and cartilage abnormality, which are reported as 1) position (being either reduced, subluxated, or dislocated), 2) stability (normal, lax, dislocatable, reducible, or not reducible), and 3) dysplasia (measured as percent of head coverage or acetabular bone inclination, as with the Graf α angle) [2–5]. The natural history of infant hips manifesting instability with stress, which are reduced at rest and which may or may not have acetabular dysplasia by sonography, is not clear, and this evokes controversy regarding management [2–17].

The aim of our study was to observe a consecutive group of infants who met the criteria of hip instability with stress, whose hips are reduced at rest and which may or may not have acetabular dysplasia by sonography (measured as percent of head coverage or acetabular bone inclination as with the Graf α angle), to determine the natural history of hip instability without treatment. We hypothesize that the acetabular index (AI) in these hips will normalize.

## Methods

With Institutional Review Board (Nemours Institutional Review Board, Jacksonville, FL) approval, we conducted a retrospective cohort case-only study on prospectively collected data involving the natural history of infant hips with specific sonographic findings: joint instability under stress but reduced at rest that may or may not have acetabular dysplasia by sonography (measured as percent of head coverage or acetabular bone inclination, as with the Graf α angle). These infants were followed by the same treating surgeon, and the data were “blindly” reviewed by a non-treating author and analyzed by a biostatistician author. The treating surgeon followed each patient with imaging using the center edge angle as a reference of resolving dysplasia.

The inclusion criteria consisted of the following: 1) consecutive cases with risk of typical DDH (no syndromic conditions), 2) infants younger than two months at first sonography, 3) sonography by the Harcke method showing hips that were reduced at rest and unstable with stress, and 4) radiographic follow up until the normalization of the AI. Swaddling was not allowed.

The exclusion criteria were hips that were dislocated (demonstrated by Ortolani positive test) or dislocatable (manifested by Barlow positive test) [18, 19]. Demographic data included sex, ages at imaging, race, delivery presentation/type of delivery, torticollis, and family history of DDH.

Dynamic sonography was performed utilizing a 12.5 MHz linear transducer in infants up to 6 months of age. Criteria for quality images included: 1) horizontal iliac line, 2) visible acetabular roof (ilium and pubis), and 3) posterior acetabulum (ischium) according to the Harcke technique [4, 5]. Measurements were made on coronal neutral images, coronal flexion images (with and without stress), and transverse flexion images (with and without stress). These measurements were repeated for first (FS) and last (LS) sonographies at time periods we considered critical for statistical analysis. The α angle and sonographical central edge angle (sCE) were measured only in coronal views. To ensure standardization of sonographic images, the rule of 1 mm accepted difference in the measurements of the acetabulum and the diameter of the femoral head was established for neutral and adduction views of the same hip. The list of measured values is presented in Additional file 1.

Acetabular index and the center-edge angle of Wiberg (CE) were measured (following Tönnis’s description) on the first anteroposterior radiographs for baseline and again on the radiograph showing normalization of the AI [20, 21]. The value for normalization of the AI was derived from the data of Tönnis and Caffey, and blended statistical analysis revealed ≤25° AI as normative [20, 21]. The age in which the hip achieved an AI ≤25° was considered the “age of acetabular index normalization.”

### Sample size and power estimations

Statistical issues considered included sample size, power estimations, and data analysis. The 26 patients who met the inclusion criteria (consecutive sampling) provided data on 48 hips, reflecting the study size. To estimate the statistical power of the study, we used “α” (type one error tolerance for 5%), effect size of 10% (0.1), and repeated measure design implying AI at the first radiography taken (mean =28.0, SD =4.0) and the radiography with “normalization of AI” value (mean =19.8, SD =3.8). With these parameters, we estimated the power to be sufficient (>80%) in determining the 10% change in AI comparing initial radiography and radiography with normalization of the AI.

### Statistical analysis

The discrete and categorical data were summarized with frequency and percentages. A normality test was performed to examine continuous data for shape, spread, and distribution. The summary statistic for the normally distributed data was achieved with the mean and standard deviation (SD), while data that violated the normality assumption were summarized with median and interquartile range (IQR). Chi-squared statistic and Fisher’s exact test were used to examine the distribution of categorical variables by the time of normalization of the AI. When the expected cell count was <2.0, Fisher’s exact test was used to adjust for the small expected cell count. To examine the predictors of AI normalization, we used a univariable unconditional logistic regression model. This model is adequate in examining predictors of a given outcome if the outcome is measured on a binary scale. The binary scale for our outcome variable (normalization of AI) was derived from the continuous variable by using cut-off points that define normal vs. late normalization. Further, we performed a multivariable logistic regression model by using a forward loading and backward elimination approach and adjusted the significance level to the numbers of variables introduced into the model following Bonferroni suggestions (0.05/n), where “n” is the number of variables entered into the multivariable model building. The rationale for the Bonferroni suggestion is to adjust for multiple comparisons in the model, which, if not addressed, will introduce measurement error into the inference.

Prior to the analysis, to determine the natural history of AI normalization, a paired sample *t*-test was used for normally distributed data; otherwise, the Wilcoxon rank-sum test was used. To examine the factors that differentiated the children with normalization of the AI at different months of age [5–12], we used repeated measures analysis of variance (RANOVA).

**Figure 1:**
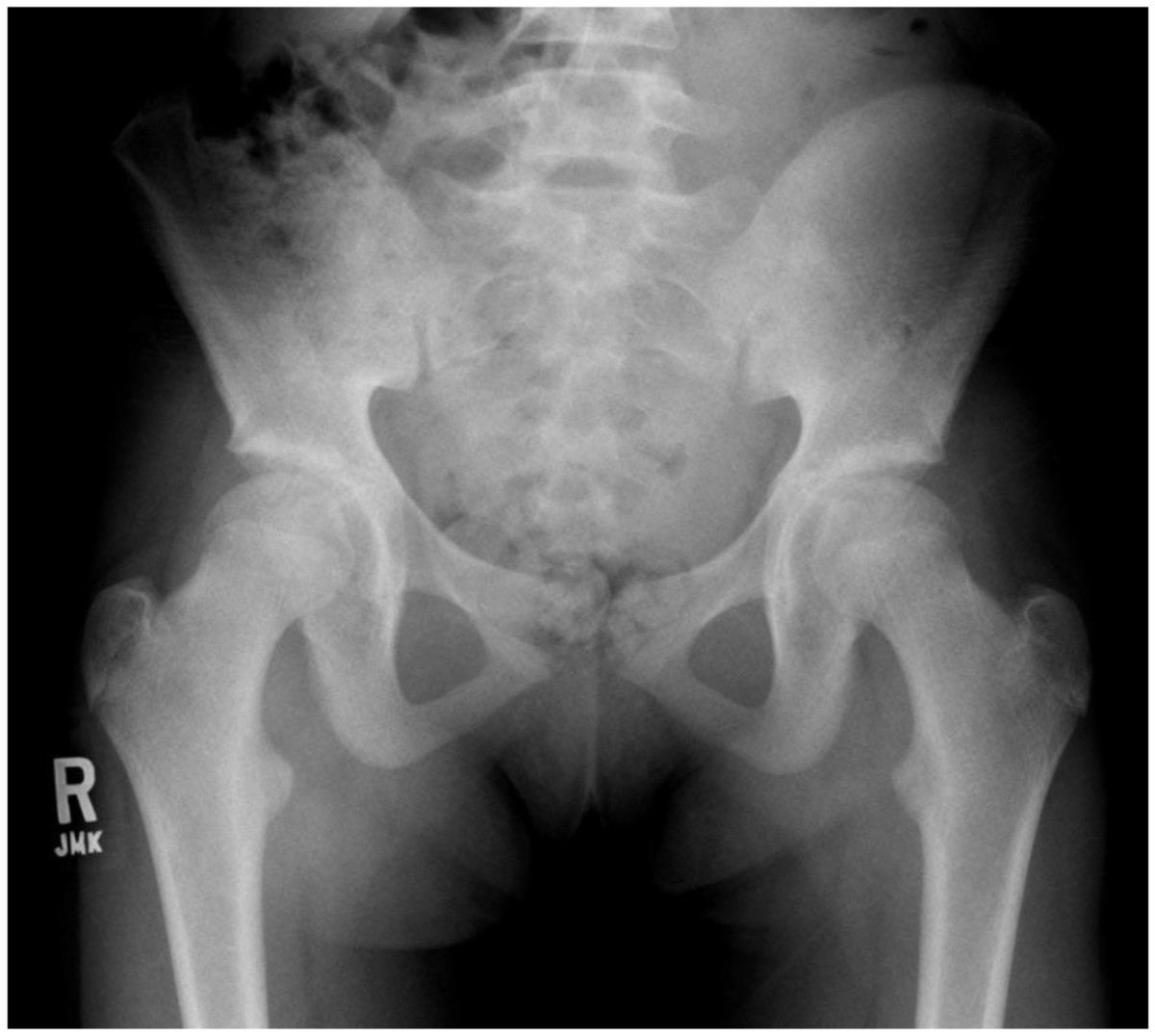
**Anteroposterior radiograph of the pelvis of the additional insightful patient at age 12 years.** The outward margin of the right acetabulum is tilted upward, and no secondary centers of ossification are present in the hip.

All tests were two-tailed, and the significance level for univariable analysis was set at <0.05. STATA statistical software, version 12.0 (STATA Corp., College Station, TX), was used to perform all the analyses.An additional insightful case (that was not part of the natural history samples) is provided to enhance discussion of our results. This 12-year-old girl presented after falling, and radiographs showed flattening of the lateral acetabular rim and delayed ossification of the secondary ossification centers of the acetabulum; the AI <25° was bilateral (Figure 1). In early infancy, she was evaluated by our institution with Harcke method sonography, which showed the hip reduced at rest and unstable with stress. She was born at term by vaginal delivery without history of breech presentation. Radiographs at 5 years of age showed a normal AI. Currently, she is still asymptomatic; however, we are concerned about the potential for arthritis in adulthood from the flattening of the lateral acetabular rim. Additionally, the senior author treated this patient’s younger sister with bilateral dislocated hips in a Pavlik harness.

## Results

This cohort case-only study consisted of 48 hips (25 patients). Sonography of one hip each in two patients did not meet inclusion criteria. No patient requested a change in treatment. Twenty-one patients were Caucasian (84%), one Asian (4%), and one Native American (4%), and, in two cases, race was undeclared (8%). The median age at the first sonography was 4 weeks (range 1–8 weeks); the second sonography was performed at a median of 8 weeks (range 8–24 weeks). The first radiograph was performed at a mean age of 21 weeks (SD = 6.4, range 12–36 weeks). The radiograph with normalized AI was performed at a median of 42 weeks of age (range 12–228 weeks). Thirty-nine hips were from girls (81.2%), and a family history of DDH was observed in 4 hips (8.3%). A total of 27 hips were breech (56.2%), 1 was transverse (2.1%), and 2 had missing data (4.2%). The type of delivery was cesarean section in 32 hips (66.7%).

Analysis of clinical and sonographic factors established two different groups of normalization of the AI of eligible infant hips: before 7 months of age (group I), and after 7 months of age (group II) (Table 1). Analysis of sonographic data concluded that three values were statistically significant (LSCNmid, LSCFaddMAD, LSCFmad) and that three values were almost significant (LSCFaddBC, LSCNmad, FSCFΔsce). Subsequent analysis of difference between groups I and II showed four sonographic factors that were statistically significant (LSCNmid, LSCFmad, LSCFaddBC, LSCFaddMID) and three factors were almost significant (FFSCFΔsce, FSTFΔa/Δd, LSTFmad) (Table 2). A combination of sonographic and clinical factors indicated the best cutoff time between groups to be at or before 7 months of age. Breech presentation (*p* = 0.013) and cesarean delivery (*p* = 0.004) statistically correlated with a slower normalization of AI (group II) (Table 3).

**Table 1:** **Potential quantitative sonographic measurement predictors of early or late acetabular index normalization**

Variable	Prevalence OR	95% CI	p	Normalized before 7 months of age (group I), number of hips =17			Normalized after 7 months of age (group II), number of hips =31		
				Median (mm)	IQR (mm)	Min-max (mm)	Median (mm)	IQR (mm)	Min-max (mm)
LSCFaddMAD	0.355	0.15-0.823	0.016	4.35	1.7	3-7.2	3.8	1	2.3-5.6
LSCNmid	31.15	1.74-558.69	0.020	1.15	0.9	0.1-1.7	1.75	0.55	1.1-2.4
LSCFmad	0.36	0.13-1.00	0.050	3.75	1.2	1.8-5.5	3.4	0.80	1.9-4.1
LSCFaddBC	12113.82	0.70-2.09	0.059	0.57	0.1	0.42-0.64	0.63	0.11	0.49-0.73
LSCNmad	0.58	0.32-1.07	0.082	3.8	0.80	2.5-6.5	3.3	1.9	-0.1-6.3
FSCFΔsce	0.92	0.85-1.01	0.092	2	10	-11-14	-2	9	-28-12
FSCNα	1.00	088-1.14	0.966	59	5	55-68	59	6	49-69
LSCNα	0.96	0.86-1.08	0.526	64	7	51-79	65	6	56-72
FSCNbc	1.06	098-1.14	0.136	54.75	10.02	36.77-70.42	58.68	12.81	43.24-90.68
LSCNbc	8.72	0.01-16345.16	0.573	61.90	22.89	50.35-78.28	65.89	13.11	54.49-85.71

Variable key: Time of measurement description (FS = first sonogram, LS = last sonogram) and code for measurement (see Additional file 1). OR = odds ratio, CI = confidence interval. All infants’ hips exhibited hip instability with stress and seated at rest with or without acetabular dysplasia on sonography. Normalization is radiographic AI ≤25°.

**Table 2:** **Sonographic measurement differences between acetabular index normalization time groups**

Sonographic measurement	Group I			Group II			p value
	Normalized before 7 months of age, number of hips =17			Normalized after 7 months of age, number of hips =31			
	Number of hips measured on sonography	Sonographic measurement		Number of hips measured on sonography	Sonographic measurement		
	n/17	Mean (mm)	SD (mm)	n/31	Mean (mm)	SD (mm)	
LSCNmid	10	0.99	0.58	16	1.7	0.42	0.001
LSCFaddMAD	14	4.66	1.16	26	3.72	0.84	0.005
LSCFmad	14	3.94	1.04	26	3.39	0.55	0.035
LSCFaddBC	14	0.56	0.07	26	0.61	0.07	0.049
FSTFΔa/ΔD	17	3.25	9.29	28	-0.68	5.38	0.078
FSCFΔsce	16	1.43	7.78	25	-3.44	9.02	0.083
LSTFmad	14	3.81	0.95	26	3.30	0.90	0.099

Variable key: Time of measurement description (FS = first sonogram, LS = last sonogram) and code for measurement (see Additional file 1). All infants’ hips exhibited hip instability with stress and seated at rest with or without acetabular dysplasia on sonography. Normalization is radiographic AI ≤25°.

**Table 3:** **Study group demographic details for individual hips**

Hip variable	Group I (normalized at 7 months of age) n =17		Group II (normalized after 7 months of age) n =31	
	number	%	number	%
Sex				
Girls	12	25.0	27	56.3
Boys	5	10.4	4	6.3
Torticolis				
No	14	29.2	25	52.0
present	3	6.3	6	12.5
Birth position				
Normal	7	14.6	11	22.9
Breech	7	14.6	20*	41.7
Transverse	1	2.1	0	0.0
No data	2	4.1	0	0.0
Type of delivery				
Physiological	5	10.4	5	10.4
C-section	7	14.6	25*	52.1
No data	5	10.4	1	2.1

Differences in acetabular normalization time for groups of infant hip instability with stress and seated at rest with or without acetabular dysplasia on sonography.

*p <0.001; all infants had hip instability under stress and seated at rest with or without acetabular dysplasia on sonography.

Since some orthopedists use the Graf α angle, some use 50% femoral head coverage (FHC), and others use the distance measured between the femoral head and the shadow of ischium bone (HID) to determine treatment, special attention was directed toward evaluation of persistence of dysplasia in regard to these parameters (Table 4). We did not find any statistically significant difference in samples or in subgroups for these measurements. These measurements were statistically significant but on a population basis. Due to the small numerical values, they are not useful on a single-case basis.

**Table 4:** **The sonographic measurement of the α angle, FHC of more than 50% and HID**

Measurement	Coronal neutral				Coronal flexion				Coronal flexion adduction			
	n		SD	range	n		SD	range	n		SD	range
α angle [°]	39	59.9*	4.9	49-69	44	59.8*	4.5	49-70	45	58*	5.1	47-72
50% FHC [%]	45	57.1^#^		36.8-90.7	50	55.3*	11.4	26.1-75.9	49	49.6*	10.6	28.3-77.3
HID [mm]		5.2^#^		2.4-8.3		5.13^#^	1.39	2.9-10.3		5.7^#^		3.4-8.6

*mean, ^#^median, 50% FHC - 50% femoral head coverage, HID - distance measured between the femoral head and the shadow of ischium bone. We did not find any statistically important difference in samples as well as in subgroups with regard to the α angle, FHC of more than 50% and HID.

## Discussion

Variability in expert-opinion recommendations for the care of infant hip instability motivated this retrospective cohort case-only study on prospectively collected data and blinded data analysis [2–17]. Prior to the development of dynamic sonography described by Harcke, hip instability in the infant could not be precisely assessed [4, 5]. Therefore, without natural history data and precise determinations of hip instability, the decision for treatment is based on the “expert opinion.” Barlow stated that 88% of the unstable hips resolved in 2 months and proposed that the remainder be treated by his malleable splint [18]. Clarke and Castaneda recommended braces for hip instability that persists greater than 6-weeks [7]. Imrie et al. presented a population of 266 breech babies in which 193 had normal sonograms at 6 weeks of age; however, radiographs of these infants at 6 months of age showed 39 hips (29% infants) with dysplasia “requiring treatment” with an orthosis [11]. Rosendahl et al. [16] followed 3613 patients in which 123 infants were treated based on clinical or sonography examination. Infants with minor dysplasia were treated only “if they were sonographically dislocated/dislocatable or borderline unstable” [16]. Infants with risk factors for DDH (breech presentation, “close family history”) had a radiograph of the hips performed at 4 to 5 months of age [16]. The Guideline of the American Academy of Pediatrics summarized 118 studies from a larger set of 624 articles and presented no specific directions for managing instability without subluxation or dislocation [13]. Kohler et al. stated that persistent hip dysplasia in radiographs at 3.5 months of age and with limitation of abduction may justify the orthotic treatment to accelerate the acetabulum development; however, every case should be considered individually [12]. Graf [10] proposed that “the gliding movement of the femoral head” is acceptable as long as the bony acetabular roof is adequate or good. However, a deficient roof (Graf type IIc and worse) may damage the hip joint if the treatment is implemented [17]. Gans et al. suggested bracing for residual acetabular dysplasia in infantile DDH if the acetabular index is ≥30° by 6 months of age [9]. Our natural history study challenges these traditions in that normalization of the AI occurred without treatment by age 7 months in 35%, 12 in 67%, 18 in 75%, 24 in 81%, and 36 in 100% of our hips. Shenton’s line was intact and the CE angle was normal on all final follow-up radiographs.

We observed two variations in ossification of the acetabulum in unstable infant hips. In group I, normalization of the AI was ≤7 months of age, which is within the expected time of ossification. In group II, normalization of the AI was delayed until after 7 months of age. The two groups were different statistically by clinical factors and sonography. The sonographic measurements of morphology (LSCNmid, LSCFmad, LSCNmad, FSCFΔsce) yielded only one statistically significant value, LSCNmid, with the others being almost significant. Measurement of instability by sonography (LSCFaddMAD) was statistically significant in predicting late AI normalization. Unexpectedly, greater instability was related to early normalization. We cannot account for this because another measurement of instability (LSCFaddBC) was almost significant in the opposite direction: i.e., greater instability was a reflection of later normalization of the AI (Table 2). Measurement of sonographic images results in very small numbered values (around one millimeter) and consequently is likely to be influenced greatly by discreet change in the position of the probe during the sonography examination; differences in muscle tone of the infant; or changes in the position of the joint from neutral to flexion or neutral to adduction, which is part of the sonographic examination protocol. Despite the strengths of the study (sample size, consecutive patient sample, rigorous methodology), there is possibility of information bias as the result of measurement parameters. Measurements were done by a single individual and although intraobserver variability was not formally calculated, we do not think that our results are likely to be driven by information bias because we performed repeated measures and reliability checks on the variables. Therefore, when the values of sonographic measurement are very small and overlapping between groups and the 95% confidence interval (CI) is unreasonably wide, implying imprecise measurement (Table 1), we do not consider these values as reliable for predicting the outcome of AI normalization.

The scope of our study cannot conclude whether or not normalization of the AI at age 3 years will lead to continued normalization until maturity. The repeated radiograph in adolescence of our “additional insightful case” showed an upward tilt of the outward portion of the acetabular rim. The lateral margin of the acetabulum is formed by the secondary ossification centers of the acetabulum, and an abnormality of this area appears to be present [6]. On the basis of our inference in this study, we suggest a third variation of acetabular ossification in DDH that is not visible in childhood but is manifest in adolescence as an upward tilt of the outer portion of the acetabular rim. This single case illustrates the dilemma orthopedists face with DDH, in that a normal AI in childhood may not mean the hip will remain normal throughout life. A similar observation was recorded by Tucci et al. in a group of 61 cases with DDH treated with a Pavlik harness in which 17% had an upward tilt of the outward portion of the acetabulum roof in a mean follow-up age of 12 years [22].

## Conclusions

In conclusion, our sample is the first natural history study of hips with instability with stress and reduced at rest (with or without acetabular dysplasia on sonography measured as percent of head coverage or acetabular bone inclination as with the Graf α angle). All our patients had a normalized AI by 3 years of age without treatment. Two patterns of growth of the acetabulum were established: group I with normal ossification, and group II with delayed ossification. This natural history study supports the supposition that treatment may be unnecessary in infant hips with instability with stress and reduced at rest (with or without acetabular dysplasia on sonography measured as percent of head coverage or acetabular bone inclination as with the Graf α angle). We propose three patterns of acetabular ossification in unstable hips of infants: (I) normal ossification, (II) delayed ossification with normalization of the AI radiographically by age 3 years, and (III) abnormality in the secondary centers of ossification of the acetabulum.

### Consent

Following the Institutional Review Board rules, written informed consent from the patient’s guardian/parent was not needed as long as the published data were deidentified and collected retrospectively without any influence on treatment.

## Electronic supplementary material

Additional file 1:The list of measured values.(DOCX 355 KB)

Below are the links to the authors’ original submitted files for images.

Authors’ original file for figure 1

Authors’ original file for figure 2

Authors’ original file for figure 3

## Abbreviations

D: Diameter of femoral head
a: Depth of acetabulum
a/D: ratio “a” to “D” (1)
AI: Acetabular index
CFadd: Sonography coronal flexion adducted image
bc: bony coverage of the acetabulum (1)
CE: Center-edge angle of Wiberg
CFadd: Sonography coronal flexion adducted image
CFn: Sonography coronal flexion neutral image
CI: Confidence interval
CN: Sonography coronal neutral image
D: Diameter of femoral head
DDH: Developmental dysplasia of the hip
FS: First sonogram
hid: Head ischium distance
HID: Head ischium distance
IQR: Interquartile range
LS: Last sonogram
MAD: Mid acetabular distance
MID: Ischium cartilage thickness
sce: Central edge angle (on sonography)
SD: Standard deviation
TF: Transverse flexion
TFadd: Transverse flexion adduction
α: α angle
Δa/ΔD: Change in ratio of change of depth of the acetabulum to the change of femoral head diameter between adduction and neutral measurement
ΔHID: Change in head ischium distance between adduction and neutral measurement
ΔMAD: Change in mid acetabular distance between adduction and neutral measurement
ΔsCE: Change in sonographic central edge angle measurement between adduction and neutral measurement
Δα: Change in α angle measurement between adduction and neutral measurement.
