# Supplementary material for: Natural history of hip instability in infants (without subluxation or dislocation): a three year follow-up
Source: BMC Musculoskelet Disord. 2014 Oct 28;15:355. doi: 10.1186/1471-2474-15-355 (PMC4236482; doi:10.1186/1471-2474-15-355)
Supplement: Supplementary file 1 — Additional file 1:The list of measured values.(DOCX 355 KB) [file 12891_2014_2306_MOESM1_ESM.docx]

**Additional file 1**

Schema of measurement conducted on sonographic images (rest and stress views) on coronal views (left image), and transverse views (right image). Each measurement is prefixed by the time code (FS = first sonogram, LS = last sonogram) - **Figure 2a and 2b.**


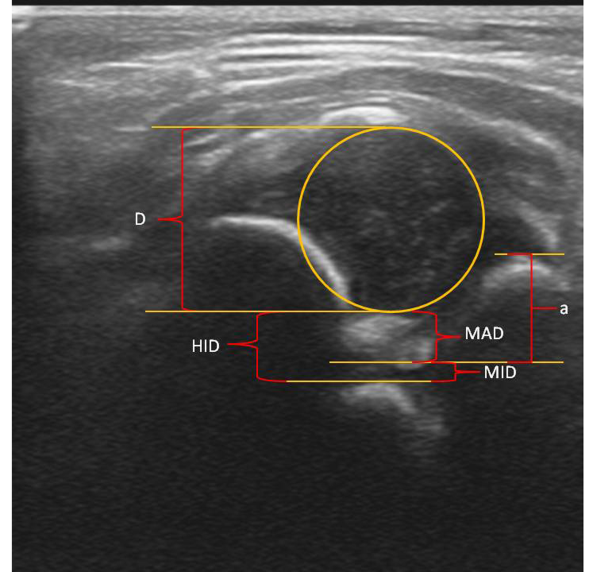

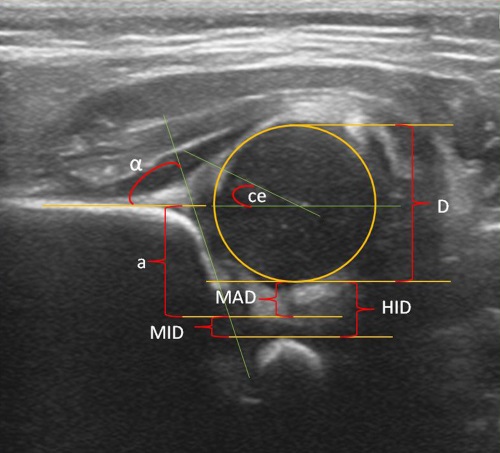


**Figure 2** Schema of measurement conducted on sonographic images (rest and stress views) on coronal views (left image, a), and transverse views (right image, b)

The measurements of sonographic images included the following: CN_D – sonography coronal neutral image diameter of femoral head, CNa – sonography coronal neutral image depth of acetabulum, CNa/D – sonography coronal neutral image ratio “a” to “D” (1), CNbc – sonography coronal neutral image bony coverage of the acetabulum (1), CNmad – sonography coronal neutral image mid acetabular distance, CNmid – sonography coronal neutral image ischium cartilage thickness, CNhid – sonography coronal neutral image head ischium distance, CNα – sonography coronal neutral image α angle, CNsce – sonography coronal neutral image sonographic central edge angle, CFnD – sonography coronal flexion neutral image diameter of femoral head, CFnA – sonography coronal flexion neutral image depth of femoral head of the acetabulum, CFnA/D – sonography coronal flexion neutral image ratio “a” to “D”, CFnBC – sonography coronal flexion neutral image bony coverage of the acetabulum, CFnMAD – sonography coronal neutral image mid acetabular distance, CFnMID – sonography coronal neutral image ischium cartilage thickness, CFnHID – sonography coronal neutral image head ischium distance, CFnα – sonography coronal neutral image α angle, CFnsCE – sonography coronal neutral image sonographic central edge angle, CFaddD – sonography coronal flexion adduction image diameter of femoral head, CFaddA – sonography coronal flexion adduction image depth of femoral head of the acetabulum, CFaddA/D – sonography coronal flexion adduction image ratio “a” to “D” (1), CFaddBC – sonography coronal flexion adduction image bony coverage of the acetabulum (1), CFaddMAD – sonography coronal adduction image mid acetabular distance, CFaddMID – sonography coronal adduction image ischium cartilage thickness, CFaddHID – sonography coronal adduction image head ischium distance, CFaddα – sonography coronal adduction image α angle, CFaddsCE – sonography coronal adduction image sonographic central edge angle, CFΔMAD – sonography coronal image change in mid acetabular distance between adduction and neutral measurement, CFΔHID – sonography coronal image change in head ischium distance between adduction and neutral measurement, CFΔα – sonography coronal image change in α angle measurement between adduction and neutral measurement, CFΔsCE – sonography coronal image change in sonographic central edge angle measurement between adduction and neutral measurement, CFΔa/ΔD – sonography coronal image change in ratio of change of depth of the acetabulum to the change of femoral head diameter between adduction and neutral measurement, CFΔa/D – sonography coronal image change in ratio of depth of the acetabulum to the femoral head diameter between adduction and neutral measurement. In the transverse flexion (TF) and transverse flexion adduction (TF add) views, the following measurements were done, similar to those described above: TFa, TFd, TFa/d, TFbc, TFmad, TFmid, TFhid, TFaddA, TFaddD, TFaddA/D, TFaddBC, TFaddMAD, TFaddMID, TFaddHID, TFΔMAD, TFΔMID, TFΔHID, TFΔA, TFΔA/ΔD, TFΔa/d, TFΔBC.
