# Supplementary figures and images for: Natural history of hip instability in infants (without subluxation or dislocation): a three year follow-up
Source: BMC Musculoskelet Disord. 2014 Oct 28;15:355. doi: 10.1186/1471-2474-15-355 (PMC4236482; doi:10.1186/1471-2474-15-355)

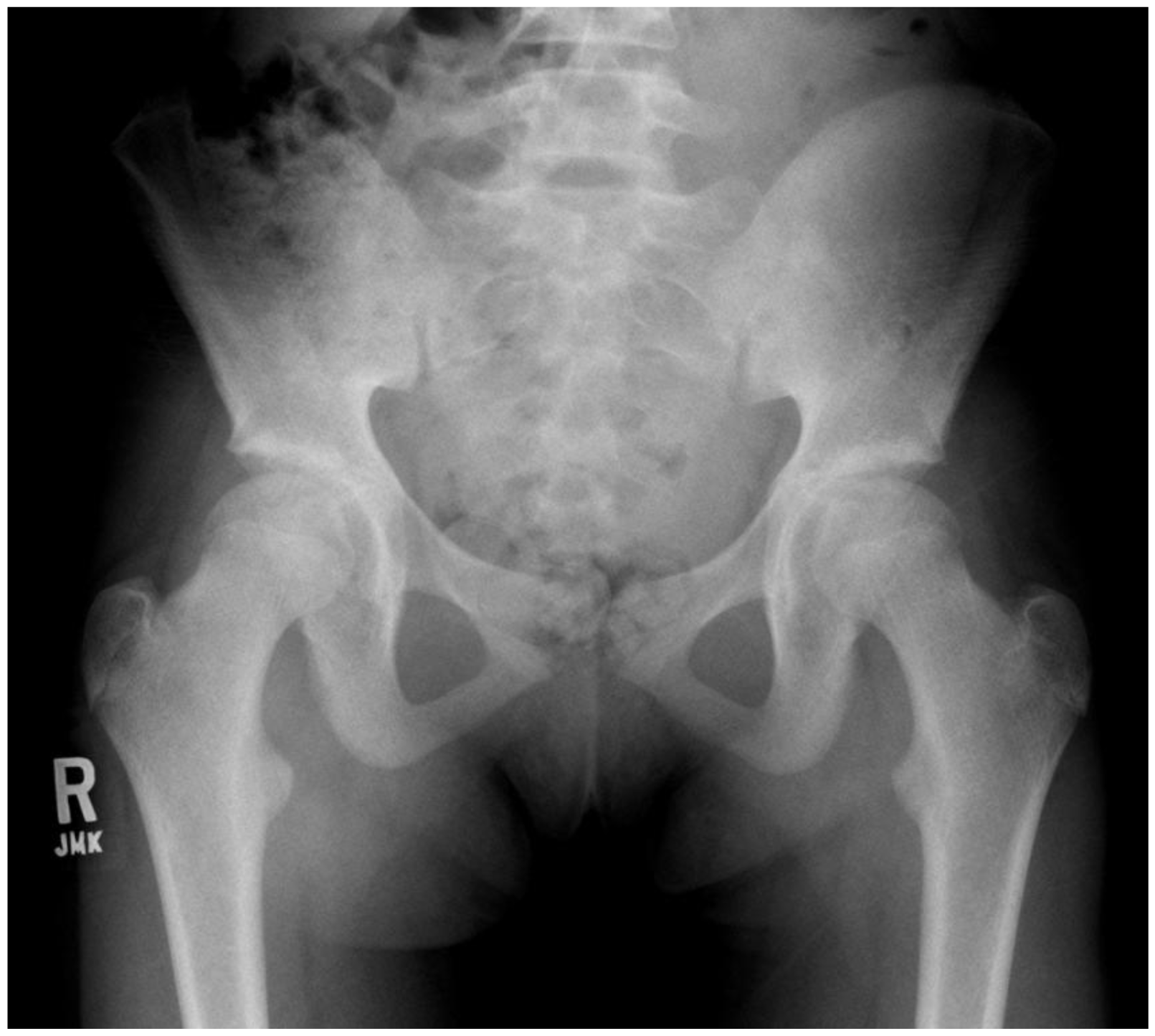

Supplement: Supplementary file 2 — Authors’ original file for figure 1 [file 12891_2014_2306_MOESM2_ESM.tiff]

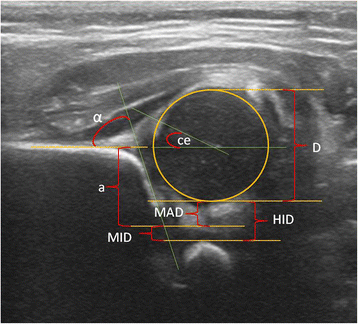

Supplement: Supplementary file 3 — Authors’ original file for figure 2 [file 12891_2014_2306_MOESM3_ESM.gif]

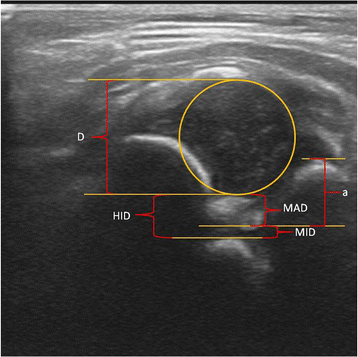

Supplement: Supplementary file 4 — Authors’ original file for figure 3 [file 12891_2014_2306_MOESM4_ESM.gif]
